# Mesenchymal stem cell–derived extracellular vesicle treatment of induced pluripotent stem cell–derived motor neurons with different amyotrophic lateral sclerosis genetic backgrounds

**DOI:** 10.4103/NRR.NRR-D-25-01790

**Published:** 2026-02-05

**Authors:** Suzy Varderidou-Minasian, Channa E. Jakobs, Svetlana Pasteuning-Vuhman, Lars Gal, Annabel Timmers, Maarten Altelaar, Magdalena J. Lorenowicz, R. Jeroen Pasterkamp

**Affiliations:** 1 Department of Translational Neuroscience, UMC Utrecht Brain Center, University Medical Center Utrecht, Utrecht University, Utrecht, The Netherlands; 2 Biomolecular Mass Spectrometry and Proteomics, Bijvoet Center for Biomolecular Research and Utrecht Institute for Pharmaceutical Sciences, University of Utrecht, Utrecht, The Netherlands; 3 Netherlands Proteomics Center, Utrecht, The Netherlands; 4 Center for Molecular Medicine, University Medical Center Utrecht, Utrecht, The Netherlands; 5 Regenerative Medicine Center, Utrecht, The Netherlands

**Keywords:** amyotrophic lateral sclerosis, C9ORF72, extracellular vesicles, FUS, induced pluripotent stem cells, mesenchymal stromal/stem cells, motor neurons, proteomics, transactive response (TAR) DNA-binding protein 43 (TDP-43)

## Abstract

Motor neurons derived from induced human pluripotent stem cells offer a powerful model to study motor neuron diseases, such as amyotrophic lateral sclerosis. While widely used, our knowledge of the proteomic changes in these models is rather rudimentary. In this study, we conducted a comparative proteomic analysis of induced pluripotent stem cell–derived motor neurons carrying amyotrophic lateral sclerosis–associated mutations in *C9ORF72*, *TARDBP*, or *FUS*. This revealed both mutation-specific and shared proteomic signatures, unveiling common and divergent disease mechanisms. Using these new insights, we then evaluated the therapeutic potential of mesenchymal stem cell–derived extracellular vesicles. These experiments showed a functional effect of mesenchymal stem cell–derived extracellular vesicles in amyotrophic lateral sclerosis-FUS motor neurons *in vitro* and their ability to reverse proteomic changes more generally in motor neurons with different amyotrophic lateral sclerosis genetic backgrounds. These findings highlight key molecular pathways involved in amyotrophic lateral sclerosis at the protein level and support the potential of mesenchymal stem cell–derived extracellular vesicles as a versatile therapeutic approach.

## Graphical abstract

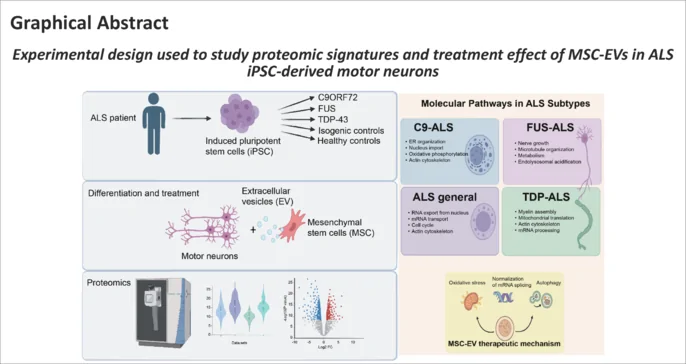

## Introduction

Amyotrophic lateral sclerosis (ALS) is an adult-onset neurodegenerative disorder characterized by progressive muscle weakness and degeneration of motor neurons (MNs) in the brain and spinal cord (Kwan and Vullaganti, 2022). Patients typically die from respiratory failure within 2–4 years following symptom onset. The disease arises from a complex interplay between environmental and genetic factors. Over 40 genes have been linked to ALS, including C9ORF72 (**~**40%) (Brown et al., 2021), FUS (**~**1%–5%) (Assoni et al., 2023), and TARDBP (**~**1%–5%) (Ma et al., 2022). Analysis of these genes has provided invaluable insight into the mechanisms underlying ALS, but translation of these findings into effective therapies remains challenging. Drugs currently approved, such as Riluzole (Wei et al., 2024) and Tofersen (Miller et al., 2020), show only modest benefits. Nevertheless, their development underscores that further insight into ALS pathogenesis is needed. Human induced pluripotent stem cell (iPSC)–derived MNs cultures are a powerful tool to study ALS pathogenesis (Pasteuning-Vuhman et al., 2021). ALS mutations have been modelled in iPSC-derived MNs (Held et al., 2023; Workman et al., 2023), but their proteomic characterization is rather incomplete. Moreover, most models are studied separately, limiting comparisons across mutation-specific and convergent molecular mechanisms. Therefore, we determined and compared proteomic signatures associated with three major ALS mutations (in *C9ORF72*, *FUS*, and *TARDBP*) in iPSC-derived MNs. These newly acquired data were used to understand the proteomic mechanisms underlying the effects of treatment with extracellular vesicles (EVs) derived from mesenchymal stromal/stem cells (MSCs) in ALS iPSC-derived MN cultures.

**Highlights**

• This study determines ALS-associated proteomic signatures in iPSC-derived MNs

• Proteomic signatures indicate common and divergent disease mechanisms in MNs

• MSC-EV treatment restores neurite growth defects in FUS-ALS MNs

• MSC-EVs restore proteomic changes in MNs carrying different ALS mutations

Several studies have demonstrated that MSCs exert neuroprotective effects in ALS models, delaying MN degeneration, reducing inflammation, and extending survival (Boucherie et al., 2009; de Munter et al., 2020; Martínez-Muriana et al., 2020; Zhou et al., 2021). Emerging evidence suggests that these therapeutic benefits are mediated by MSC-EVs. EVs are heterogeneous membrane-bound vesicles that transport proteins, RNA, lipids, and carbohydrates, between cells (Varderidou-Minasian and Lorenowicz, 2020b). For example, MSC-EVs selectively promote neurite outgrowth in cortical neuron cultures and modulate neural plasticity (Ma et al., 2020; Fuloria et al., 2021). To further define the mechanism of action of MSC-EVs in the context of ALS, we treated mutant ALS iPSC-derived MNs with MSC-EVs and studied cellular and proteomic changes.

Thus, although widely used, our knowledge of the proteomic changes in iPSC-derived motor neuron models is rather rudimentary. Therefore, in this study, we determined and integrated the altered proteomic profiles of MNs carrying different ALS-associated mutations. We subsequently exploited these data to study the beneficial effect of MSC-EV treatment in ALS-FUS MNs *in vitro* and the ability of MSC-EVs to reverse proteomic changes more generally in MNs with different ALS genetic backgrounds.

## Methods

### Cell culture

#### Induced pluripotent stem cell lines and culture

The iPSC lines (**Additional Table 1**) used in this study were reprogrammed from skin fibroblasts with informed consent. iPSC work was approved by the Toetsingscommissie biobank UMC Utrecht (deel biobank 16-436, 30-03-2017). C9ORF72 and healthy control (HC) lines used were as described by (De Decker et al., 2024; van der Geest et al., 2024). iPSCs were cultured on Geltrex-coated dishes (Cat# A1413202, Gibco, Carlsbad, CA, USA), passaged weekly with Trypsin-ethylenediaminetetraacetic acid (EDTA; Cat# 25200072, Life Technologies, Carlsbad, CA, USA), and maintained in StemFlex^TM^ medium (Cat# A3349401, Life Technologies) with Y-27632 for the first 24 hours. Cells were frozen in FBS (Cat# F7524, Sigma, St. Louis, MO, USA) with 10% DMSO and stored in liquid nitrogen. Thawing was done at 37°C followed by washing with cold Dulbecco’s Modified Eagle Medium (DMEM)/F12 (Cat# 11320074, Gibco).

**Additional Table 1:** Information of patient and control lines used for this study

Cell line	Name in lab	Gender	Age of Biopsy	Virus used	Genotype	Where made	Reference	ALS pathology in iPSC-motor neurons
**C9-ALS**								
**C9-1**	CS52iALS-C9n6	Male	45	Episomal Plasmid	C9orf72 Hexanucleotide repeat expansion (6-8kb)	Cedars Sinai	https://biomanufacturing.cedars-sinai.org/product/cs52ials-c9nxx/; Sareen et al. (Science Translational Medicine, 2013)	RNA foci selectively in C9-ALS motor neurons; Increased dipeptide repeat (DPR) proteins and disrupted nucleocytoplasmic transport
**C9-2**	CS29iALS-C9n1	Male	47	Episomal Plasmid	C9orf72 Hexanucleotide repeat expansion (6-8kb expanded allele)	Cedars Sinai	https://biomanufacturing.cedars-sinai.org/product/cs29ials-c9nxx/; Sareen et al. (Science Translational Medicine, 2013)	RNA foci selectively in C9-ALS motor neurons; Increased dipeptide repeat (DPR) proteins and disrupted nucleocytoplasmic transport
**C9-3**	C902-02	Female	62	retrovirus	C9orf72 (clone 2 from patient C902)	Talbot Lab Oxford	https://doi.org/10.1002/stem.2388	Ca^2+^ dysregulation, endoplasmic reticulum stress, loss of proteostasis. Impaired mitochondrial Ca^2+^ uptake and glutamate excitotoxicity
**iCTRL**								
**C9-1-iso**	CS52iALS-C9n6.ISOC3	Male	46	Used CRISPR/Cas9	C9 mutation corrected via CRISPR Cas9	Cedars Sinai	https://biomanufacturing.cedars-sinai.org/product/cs52ials-c9n6-isoxx/	
								
								
**C9-2-iso**	CS29iALS-C9n1.ISOT2RB4	Male	47	Used CRISPR/Cas9	C9 mutation corrected via CRISPR Cas9	Cedars Sinai	https://biomanufacturing.cedars-sinai.org/product/cs29ials-c9n1-isoxx/	
								
**C9-3-iso**	59.1 (ISO CTRL C902-02)	Female	62	Used CRISPR/Cas9	C9orf72 most repeats deleted but locus intact (2 repeats left).	Talbot Lab Oxford	https://doi.org/10.1002/stem.2388	
**Healthy controls (HC)**								
**HC-1**	PZ1-5	Male	62	Sendai virus	Healthy ctrl	UMC Utrecht	https://doi.org/10.1186/s40478-024-01852-6	
**HC-2**	929c4	Female	60	Sendai virus	Healthy ctrl	UMC Utrecht	made at UMC Utrecht Brain Center MIND facility (manuscript in submission)	
**HC-3**	OH3.1	Male	49	Lentivirus	Healthy ctrl	UMC Utrecht	10.1038/s41467-018-06684-2 (iPSC 5)	
**TDP-ALS**								
**TDP-1**	TDP0102-M337V	Male	61	Sendai	TDP43 I383T mutation	University of Oxford	10.1016/j.stemcr.2020.03.023	High glutamate-induced Ca^2+^ release, impaired mitochondrial Ca^2+^ uptake and glutamate excitotoxicity
**TDP-2**	CiRA 00024	Female	60	retrovirus	TDP43 M337V mutation	Riken Japan	10.2169/internalmedicine.49.2915; DOI: 10.1126/scitranslmed.3004052	Cytoplasmic aggregates, shorter neurites
**TDP-3**	M337V.2	Male	59	Episomal reprogramming	TDP43 M337V mutation	University of Edinburgh	https://doi.org/10.1073/pnas.1202922109	Cytoplasmic accumulation of TDP-43, increased cell death
**FUS-ALS**								
**FUS-1**	FUS2	Male	26	Lentivirus	R495QfsX527 Frameshift mutation	Hannover Germany	https://doi.org/10.1002/stem.2354	Hypoexcitability in induced pluripotent stem cell-derived motor neurons, ion channel imbalances with lower sodium to potassium (Na^+^/K^+^) ratios
								
**FUS-2**	FUS 2/2	Female	71	Sendai virus	R521H FUS Heterozygous point mutation	Leuven	10.1038/s41467-017-00911-y	Cytoplasmic accumulation of FUS, hypoexcitability, mitochondrial and endoplasmic reticulum vesicle axonal transport defects.
								
**FUS-3**	FUS 3/1	Male	17	Sendai virus	P525L FUS Heterozygous point mutation	Leuven	10.1038/s41467-017-00911-y	Cytoplasmic accumulation of FUS, hypoexcitability, mitochondrial and endoplasmic reticulum vesicle axonal transport defects.
**iCTRL**								
**FUS-1-iso**	FUS2 isogenic control	Male	26	CRISPR/Cas9	Wild type FUS	Hannover Germany	https://doi.org/10.1002/stem.2354	
**FUS-2-iso**	FUS CO2/2	Female	71	Made from FUS2/2, company Cell System	FUS R521H	Cell System	10.1038/s41467-018-06111-6	
**FUS-3-iso**	FUS CO3/3	Male	17	Made from FUS3/3, company Cell System	FUS P525	Cell System	10.1038/s41467-018-06111-6	

Lines were used up to passage 55, and pluripotency was confirmed using the StemLight Pluripotency Antibody Kit (Cat# 9656S, Cell Signaling, Danvers, MA, USA), the STEMdiff^TM^ Trilineage Differentiation Kit (Cat# 05230, Stem Cell Technologies, Vancouver, BC, Canada), and quantitative reverse transcription-polymerase chain reaction (qRT-PCR). Media were refreshed every other day, and all cultures were routinely tested for mycoplasma using the MycoAlert kit (Cat# LT07-318, Lonza, Basel, Switzerland). Cell viability of MNs with different genetic backgrounds was assessed using the Trypan Blue exclusion assay.

#### Motor neuron differentiation

Spinal MN differentiation was performed according to the methods of Du et al. (2015). Briefly, iPSCs were dissociated with 1 mg/mL Dispase (Stem Cell Technologies) and plated on Geltrex-coated plates. Basal medium was used throughout the differentiation process which consisted of a 1:1 mixture of DMEM/F12 and Neurobasal media (Gibco), supplemented with 1× sodium pyruvate, 100 µM non-essential amino acids, 1× GlutaMAX, 1× penicillin/streptomycin, 1× N2 supplement (Cat# 17502001, Gibco), and 1× B27 supplement (Cat# 17504044, Gibco). After 2 days, medium was changed with basal medium supplemented with ascorbic acid (0.4 mM) (Sigma, Cat# A4403), LDN193189 (0.2 μM), CHIR99021 (3 mM) (Cat# SML1046, Sigma), and SB431542 (10 μM) (Cat# AXON1661, Axon Biochemicals, Groningen, the Netherlands) for 6 days, with medium changes every other day. At days *in vitro* (DIV) 7, medium was changed to basal medium supplemented with ascorbic acid (0.4 mM), LDN193189 (0.2 μM) (Cat# 130-106-540, Miltenyi Biotec B.V. (Netherlands)), CHIR99021 (1 μM), SB431542 (10 μM), retinoic acid (RA) (200 nM) (Cat# R2625, Sigma), Purmorphamine (0.5 mM), and cAMP (0.5 µM) for another 6 days, with daily medium changes. At DIV12, cells were dissociated with Accutase (Cat# SCR005, Millipore, Burlington, MA, USA) and cultured in suspension with ascorbic acid (0.4 mM), cAMP (0.5 μM), RA (500 nM), and purmorphamine (0.1 μM) for 6 days, with medium changes every other day. At DIV18, cells were dissociated, plated on poly-D-lysine (Cat# P0899, Sigma) and Laminin-coated coverslips (Cat# 23017-015, Thermo Fisher, Waltham, MA, USA), and cultured in basal medium with ascorbic acid (0.4 mM), cAMP (1 μM), brain-derived neurotrophic factor (Cat# 78005, Stem Cell Technologies), GDNF (Cat# 78058, Stem Cell Technologies), CNTF (10 ng/mL, Cat# 78010, Stem Cell Technologies), and BrainXell supplement (BX-2000). Medium was changed twice a week. Characterization and treatments were performed at DIV24–25.

#### Mesenchymal stromal/stem cell culture

Human bone marrow samples were obtained with informed consent and approval from the Dutch Central Committee on Research Involving Human Subjects (CCMO; Biobanking bone marrow for MSC expansion, approval no. NL41015.041.12, 17-01-2013). Bone marrow was isolated through density gradient centrifugation using Lymphoprep (Cat# 1856, Abbott, Chicago, IL, USA), and MSCs were isolated by plastic adherence, as previously described (Mattei et al., 2025). MSCs were cultured in alpha-modified Eagle’s medium (Cat# 12571089, Gibco) supplemented with 1% L-Glutamine (Cat# 25030024, Gibco), 100 U/mL penicillin, 100 µg/mL streptomycin (Cat# 15140122, Gibco), 5% human platelet lysate, and 10 U/mL heparin (Prins et al., 2009). Medium was changed every 3 days. MSCs were washed with PBS, detached using 0.05% Trypsin-EDTA, and cultured up to passage 3 in 175 cm^2^ culture flasks.

### Extracellular vesicle isolation

EVs were isolated from MSC culture medium as described by Warmink et al. (2023). MSCs were cultured for 48 hours in EV-depleted medium (centrifuged at 100,000 × *g* overnight). The medium was then centrifuged in steps: at 200 × *g* (10 minutes), 500 × *g* (10 minutes), and 10,000 × *g* (45 minutes). EVs were pelleted by ultracentrifugation at 100,000 × *g* for 16 hours at 4°C using an SW32Ti rotor (Beckman Coulter, Inc., Brea, CA, USA). After washing in PBS with 0.5% BSA, EVs were pelleted again overnight at 100,000 × *g* using an SW60 rotor (Beckman) and stored at –80°C. For MN treatment, EVs from 4 × 10^6^ MSCs (or 3 × 10^8^ particles) were added per well in a 24-well plate for 2 days. For neurite outgrowth assays, MNs from FUS-ALS MNs and iCTRL (FUS-3 and FUS-3-iso) were used. For mass spectrometry, all samples (C9-ALS, FUS-ALS, TDP-43-ALS, iCTRL and HC) were used.

For CD9 and CD63 expression analysis, EVs were further enriched using a sucrose density gradient. The EV pellet was resuspended in 250 μL PBS with 2.5 M sucrose and layered with 15 successive 250 μL layers of 20 mM Tris-HCl (pH 7.4) containing sucrose concentrations ranging from 2 M to 0.4 M. The gradient was ultracentrifuged at 200,000 × *g* for 16 hours at 4°C. After centrifugation, 250 μL fractions were collected, and the sucrose density was measured using a refractometer.

### Nanoparticle tracking analysis

The size distribution and quantification of isolated EVs were determined using Nanosight NS500 (Marvern, Worcestershire, UK) with the following settings: camera level: 13, slider shutter: 1232, slider gain: 219, detection: 5, autoblur mode for the analysis. Samples were diluted 100, 300, 500, 700, and 1000 times with PBS to reach an optimal concentration for instrument linearity. Data was processed using Nanosight NS500 (Malvern Panalytical, Malvern, UK) NTA Software v3.1.54 employing the finitetracklength adjusted algorithm with 10 nm bin widths. Size distribution and concentrations were calculated using build-in software.

### RNA isolation, cDNA synthesis, and quantitative reverse transcription-polymerase chain reaction

MNs were lysed using TRIzol (Thermo Fisher) and RNA extracted with the RNeasy Mini Kit (Qiagen, Hilden, Germany), including on-column DNase treatment. RNA concentration and purity were measured with a NanoDrop 2000. Intron-spanning primers were designed with PrimerBLAST (**Additional Table 2**). cDNA synthesis was done using the SuperScript IV Reverse Transcriptase Kit (Thermo Fisher).

**Additional Table 2:** qPCR primers used in this study

Gene	Primer (5'-3')
*NANOG*	F: GCCTGTGATTTGTGGGCCTGA R: GTGGAAGAATCAGGGCTGTCCTG
*SOX2*	F: CGAGGGAAATGGGAGGGGTGC R: TGCAGCTGTCATTTGCTGTGGGT
*PAX6*	F: AGTTCTTCGCAACCTGGCTA R: ATTCTCTCCCCCTCCTTCCT
*ISL1*	F: AAGGACAAGAAGCGAAGCAT R: TTCCTGTCATCCCCTGGATA
*HB9*	F: TGCCTAAGATGCCCGACTT R: TTGAGCTTGAACTGGTGCTC
*CHAT*	F: GACGTCTGACGGGAGGAG R: TCAATCATGTCCAGCGAGTC
*RBFOX3*	F: GACGCAATGGTTCAGCCTTTT R: GCGTACTTCCGTAGAGTGTCAG
*VACHT*	F: CTGCTAGTGAACCCCTTGAGC R: CAGGACTGTAGAGGCGAACAT
*SYNAPSIN*	F: AGTTCTTCGGAATGGGGTGAA R: CAA ACT GCG GTA GTC TCC GTT
*NFH*	F: GCAGTCCGAGGAGTGGTTC R: CGCATAGCGTCTGTGTTCA
*MAP2*	F: TGCCTCAGAACAGACTGTCA R: GGCTCTTGGTTACTCCGTCA

F: Forward; R: reverse

qPCR was performed with 2x FastStart Universal SYBR Green Master (Roche, Basel, Switzerland) on a QuantStudio 6 Flex System (Thermo Fisher), following the standard PCR program. Samples were analyzed in duplicate, and fold changes were calculated using the 2^–ΔΔCT^ method, normalized to *GAPDH*, *BETA-ACTIN*, and *TBPQ*. Data analysis was performed with GraphPad Prism (V10, GraphPad Software, San Diego, CA, USA, www.graphpad.com).

### Immunocytochemistry

MNs were fixed in 4% paraformaldehyde for 20 minutes at 4°C and permeabilized with 0.1% Triton X-100 in PBS for 5 minutes. Blocking was done with 2% BSA, 0.1% Triton X-100, and 5% goat serum in PBS for 30 minutes. Primary antibodies were incubated overnight at 4°C: ISL LIM homeobox 1 (ISL1; Cat# AB2314683, 1:400, anti-mouse, Developmental Studies Hybridoma Bank (DSHB), Iowa City, IA, USA), choline acetyltransferase (CHAT; Cat# AB144P, 1:50, anti-goat, Millipore), SMI-32 (neurofilament H, NFH; Cat# AB531793, 1:1000, anti-mouse, DSHB), motor neuron and pancreas homeobox 1, formerly HB9 (HB9; Cat# AB2145209, 1:50, anti-mouse, DSHB), class III β-tubulin (TUJ1; Cat# 801201, 1:500, anti-mouse, Biolegend, San Diego, CA, USA), Caspase-3 (Casp-3; Cat# AB3623, 1:1000, anti-rabbit, Millipore) and NANOG (Cat# 9656S, 1:1000, anti-rabbit, Cell Signaling). After washing, cells were incubated with Alexa Fluor-conjugated secondary antibodies (donkey anti-mouse Alexa Fluor, 488 (Cat# A21202), donkey anti-mouse Alexa Fluor, 568 (Cat# A31570), donkey anti-rabbit Alexa Fluor, 488 (Cat# A21206), and donkey anti-rabbit Alexa Fluor, 568 (Cat# A10042) (1:500, Thermo Fisher) for 1 hour at room temperature, followed by 4′,6-diamidino-2-phenylindole (DAPI; Cat# H3570, Thermo Fisher) counterstaining for 10 minutes. Cells were mounted with FluorSave (Millipore) and imaged by epifluorescence microscope A1 (Zeiss Axioscope, Jena, Germany).

### Western blotting

Cell and EV pellets were lysed in RIPA buffer (25 mM Tris-HCl, 150 mM NaCl, 1% NP40, 1% NaDOC) with 0.1% SDS, protease inhibitors (Roche), and NuPAGE loading buffer (Invitrogen), then heated at 95°C for 5 minutes. Protein concentration was measured using the microBCA Protein Assay Kit (Thermo Fisher). For sodium dodecyl sulfate polyacrylamide gel electrophoresis (SDS-PAGE), proteins were separated, transferred to a polyvinylidene difluoride membrane (Cat# 10600021, GE Healthcare, Chicago, IL, USA), and blocked with 5% BSA for 1 hour. Membranes were incubated overnight at 4°C with primary antibodies: CD9 (Cat# 312105, 1:1000, anti-mouse, Biolegend), CD63 (Cat# 10628D, 1:1000, anti-mouse, Thermo Fisher), tumor susceptibility gene 101 (TSG101; Cat# PA5-31260, 1:500, anti-rabbit, Thermo Fisher), Calnexin (Cat# AB75801, 1:1000, anti-rabbit, Abcam, Cambridge, MA, USA). After washing, blots were incubated with secondary antibodies IRDye 800CW goat anti-rabbit (Cat# 926-3221, 1:1000, LI-COR Biosciences, Lincoln, NE, USA) or IRDye 680CW goat anti-mouse (Cat# 926-68070, 1:1000, LI-COR Biosciences) in 5% BSA for 1 hour at room temperature (RT). Immunoreactivity was detected using Image Studio Lite (LI-COR) software.

### Proteomics

#### Sample preparation

Samples were collected and lysed with sodium deoxycholate (SDS) lysis buffer containing 1% SDC, 10 mM tris(2-carboxyethyl)phosphine hydrochloride, 40 mM chloroacetamide, and 100 mM TRIS, pH 8.0, supplemented with protease inhibitor cocktail. The lysate was sonicated using a Bioruptor (Diagenode, Liège, Belgium) and centrifuged at 2500 × *g* for 10 minutes at 4°C. A total of 20 µg protein per sample was digested overnight with trypsin (1:50 protein-to-enzyme ratio) at 37°C. The digestion was quenched with 2% formic acid (FA). The resulting peptides were centrifuged at 20,000 × *g* for 10 minutes and vacuum-centrifuged to dryness. The digestion was acidified and desalted using C18 cartridges on the AssayMap BRAVO Platform (Agilent Technologies, Santa Clara, CA, USA). Samples were dried and resuspended in 50 mM triethylammonium bicarbonate at a final concentration of 5 mg/mL prior to high-resolution tandem mass spectrometry (LC-MS/MS) analysis.

#### Mass spectrometry analysis

Mass spectrometry was performed in the Orbitrap Q-Exactive HF-X mass spectrometer (Thermo Scientific) coupled to reversed-phase nano-LC-MS/MS using Ultimate 3000 ultrahigh-performance liquid chromatography. Peptides were first separated on a 50 cm reversed-phase column (Agilent Poroshell EC-C18, 2.7 µm, 50 cm × 75 µm, in-house packed) and eluted using a linear gradient with buffer A (0.1% FA) and buffer B (80% acetonitrile, 0.1% FA). Peptides were eluted over a 120-minute gradient from 13% to 44% buffer B at a flow rate of 300 nL/min. A column wash and re-equilibration step was followed by 55 minutes of total data acquisition time. The Q-Exactive HF-X mass spectrometer was operated in data-independent acquisition (DIA) mode, with a m/z range of 345-1650. Full-scan spectra were recorded at a resolution of 120,000 with an automatic gain control (AGC) target of 3 × 10^6^ with a maximum injection time of 100 ms. The normalized collision energy was set to 27%, and the default charge state was set to 3.

#### Data processing

The protein sequence for *Homo sapiens* (UP000005640) was downloaded from UniProt (uniport.org/proteomes/UP000005640) Raw files were processed using DIA_NN (version 1.8.1, github.com/vdemichev/DiaNN) to generate spectral libraries with the following parameters: Trypsin digestion (up to 2 missed cleavages), precursor m/z range 300–1800, fragment ion m/z 200–1800, precursor charge 1–4, and peptide length 7–30 amino acids. N-terminal methionine excision and cysteine carbamidomethylation were enabled, with default settings (1% false discovery rate (FDR) and match between runs). The resulting pg_matrix.tsv file was used for analysis.

Batch effect correction was performed using the ComBat algorithm (Johnson et al., 2007) via the R script combat_corrected. Raw data were log-transformed, normalized, and corrected for batch effects while preserving biological variation. Corrected data were used for downstream analyses.

#### Data visualization

Normalized protein and peptide abundances were extracted and further analyzed using the open software PERSEUS environment (maxquant.org/perseus) for statistical and bioinformatics analysis and to generate the plots and figures. For several plots, GraphPad Prism (V10) was used. GO analysis was performed using Enrichr (Maayanlab.com). PANTHER pathway classification system (http://www.pantherdb.org/pathway/) was used for protein class analysis. Protein-protein interaction networks were constructed using the STRING database (string-db.org) and visualized in Cytoscape (cytoscape.org).

### Statistical analysis

Two samples (C9-1 B1R1 and C9-1_iso B1R1) were excluded due to insufficient sample identification. Volcano plots were generated for the specified conditions, with proteins classified as up- or downregulated based on differentially expressed criteria (Varderidou-Minasian et al., 2020a): a *P*-value ≤ 0.1 and a fold change ≥ 1.3. These thresholds were selected to balance statistical significance with biological relevance based on the data distribution. To study ALS mutation-specific alterations, C9-ALS, FUS-ALS, or TDP-ALS samples were categorized in one group and compared with HC or isogenic (iso) control (iCTRL) samples. For general ALS-related alterations, C9-ALS, FUS-ALS, and TDP-ALS samples were categorized into a single group and compared to HC samples.

GraphPad Prism was used for all statistical analyses. For each biological condition, data were obtained from a minimum of two independent differentiations. Neurite length measurements are presented as group mean ± standard error of the mean (SEM), and statistical significance was assessed using a two-tailed Student’s *t*-test. For Casp-3 analysis, statistical comparisons were performed using a one-way analysis of variance followed by Tukey’s multiple comparison *post hoc* test.

## Results

### Proteomics analysis of induced pluripotent stem cell–derived motor neurons with different amyotrophic lateral sclerosis backgrounds

**Figure 1:**
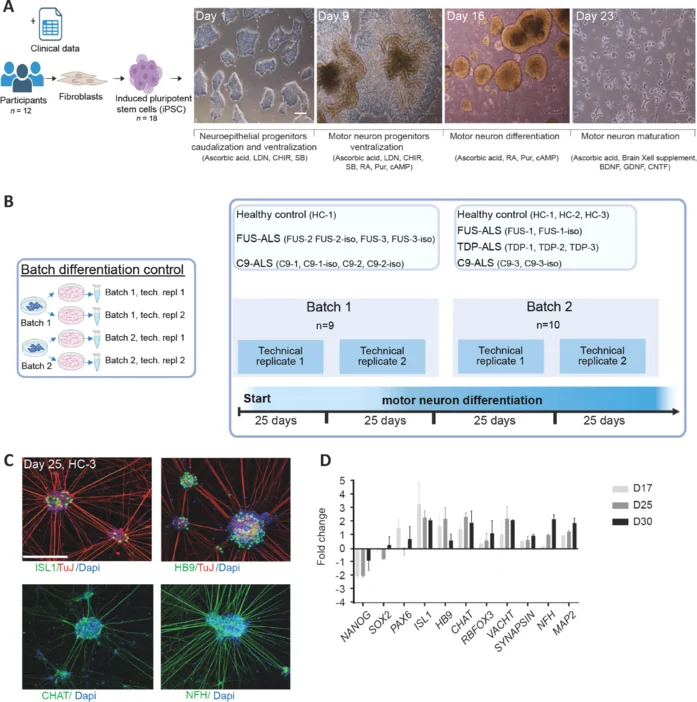
Differentiation and characterization of iPSC-derived motor neurons. (A) Schematic overview of the differentiation of iPSCs into motor neurons (MNs). Healthy control (HC), C9-ALS (carrying C9ORF72 repeat expansions), C9 isogenic control (C9-iso), FUS-ALS, FUS-iso, and TDP-ALS lines were differentiated into DIV25 MNs. (B) Schematic overview of sample distribution and batches. A total of 18 iPSC lines were divided into two batches (Batch 1: *n* = 9, Batch 2: *n* = 10), with one healthy control line (HC-1) included in both batches. Each batch was differentiated two times (technical replicate) to control for technical variability. (C) Representative images of DIV25 MNs (HC-3) immunostained for 1) ISL1 (in green) and TuJ (in red), 2) HB9 (in green) and TuJ (in red), 3) CHAT (in green), and 4) NFH (in green). Dapi is in blue. Scale bar: 250 µm. (D) mRNA expression levels of iPSC-derived MNs differentiated for 17, 25, and 30 days. The analysis includes expression of neural stem cell markers (NANOG, SOX2, and PAX6), MN markers (ISL1, HB9, CHAT, RBFOX3, VACHT, and NFH), and mature MN markers (SYNAPSIN and MAP2). Graph shows fold change values relative to gene expression in iPSCs, comparing two independent differentiations of iPSC-derived MNs (HC-3; *n* = 2). Values > 1 indicate upregulation in MNs whereas values < 1 indicate downregulation relative to expression in iPSCs. ALS: Amyotrophic lateral sclerosis; CHAT: choline acetyltransferase; DAPI: 4′,6-diamidino-2-phenylindole; DIV: days *in vitro*; HB9: motor neuron and pancreas homeobox 1, formerly HB9; iPSC: induced pluripotent stem cell; ISL1: ISL LIM homeobox 1; NFH: neurofilament H; TuJ: class III β-tubulin.

To study the effect of ALS-associated gene mutations at the protein level, human MNs were generated from previously reported iPSCs carrying genetic defects in C9ORF72, the most common genetic cause of ALS, or carrying ALS-associated mutations in the RNA-binding proteins FUS or transactive response (TAR) DNA-binding protein 43 (TDP-43). Neuronal cultures derived from these lines have previously been shown to display ALS pathological features (e.g., dipeptide repeat proteins, RNA foci, and protein mislocalization) and various cellular phenotypes (e.g., altered excitability and cell death) (Ramic et al., 2021; Scaber et al., 2024). All iPSC lines were generated from fibroblasts and were subjected to karyotyping. Detailed information regarding the iPSC lines is shown in **Additional Table 1**. Functional spinal lower MN were generated using a modified version of a previously published protocol (Du et al., 2015; Zelina et al., 2024; **Figure 1A**). iPSCs were differentiated into MNs in six-well plates, with all iPSC lines (*n* = 18) organized into two different batches, each containing 9–10 lines (**Figure 1B**). One HC line was included in both batches (HC-1). Each batch contained a mix of HC, ALS, and iCTRL. To assess variability during the differentiation process, the iPSC lines within each batch were subjected to two independent differentiations (i.e., two technical replicates). All lines were successfully differentiated into MNs within 25 days (DIV25), in line with previous studies (Dafinca et al., 2020). Cultures contained HB9- and ISL1-positive MN, in addition to expression of CHAT and SMI32/NFH, as shown by immunofluorescence analysis (**Figure 1C** and **Additional Figure 1A**). These markers were further confirmed by qRT-PCR showing HB9, ISL1, CHAT, and NFH expression at DIV17, DIV25, and DIV30 (**Figure 1D**). Markers of iPSCs (NANOG, SOX2) and neural progenitors (PAX6) were low, whereas several other (motor) neuron markers displayed increased expression during differentiation. No obvious differences in the expression of MN markers or cell viability were detected between HC and ALS cultures at DIV25, consistent with a previous study (Workman et al., 2023) (**Figure 1D** and **Additional Figure 1B**).

To resolve the proteome changes caused by different ALS-associated mutations in iPSC-derived MNs, LC-MS/MS analysis was performed on the different samples outlined above. Up to 6209 proteins were identified with an FDR of 1%, with data demonstrating normal distribution across samples and replicates (**Additional Figure 2A** and **B**). The different number of proteins identified per sample, ranging from 2833 to 5478 proteins, is likely due to variability in sample preparation. We removed two samples (C9-1 B1R1 and C9-1-iso B1R1) due to insufficient protein identification by mass spectrometry. Annotation of the proteins revealed a wide variety of protein classes, including enzymes, membrane proteins, cytoskeletal proteins, and gene-specific transcriptional regulators (**Additional Figure 2C**). iPSC markers such as SOX2 and OCT4 were not detected, but samples showed strong expression of neuronal markers (e.g., GAP43, NEFL, MAP2, and NCAM1). Further, FUS and TDP-43 were consistently expressed, whereas C9ORF72 was not detected, possibly due to low abundance (**Additional Figure 2D**).

To evaluate overall protein expression patterns across lines and batches, principal component analysis (PCA) was performed. The first principal component (PC1) accounted for 11.8% of the variation and PC2 for 7.1% (**Additional Figure 3A**). The raw proteomic data revealed a clear segregation of samples according to batches, indicating substantial batch-associated variance. To address this, batch effect correction was performed using the R script combat_corrected, based on the ComBat algorithm. Following correction, the PCA demonstrated improved clustering of samples according to their biological conditions rather than batch origin (**Additional Figure 3B**), confirming effective removal of batch-related variation. To further assess data consistency across experiments, one healthy control line (HC-1) was included in all batches and replicates. Pearson correlation analysis of this control confirmed high concordance between samples, indicating that data quality and reproducibility were maintained across batches and replicates (**Additional Figure 3C**). These observations align with findings from previous, large-scale studies of iPSC-derived MNs from ALS patients (Workman et al., 2023). Collectively, these results highlight the robustness of the differentiation protocol and reveal inter-individual variability in the proteomic landscape of iPSC-derived MNs.

### Amyotrophic lateral sclerosis mutation-specific proteomic signatures in induced pluripotent stem cell-derived motor neurons

**Figure 2:**
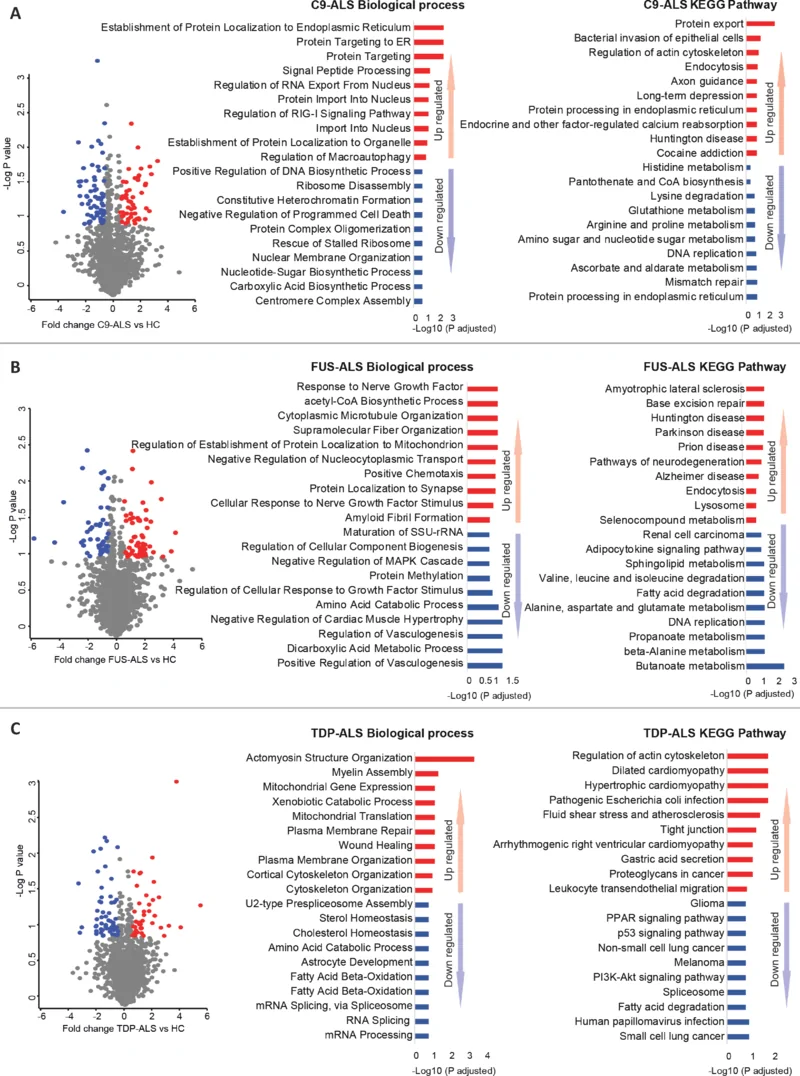
ALS mutation-specific proteomic changes in iPSC-derived motor neurons. Volcano plots showing differentially expressed proteins in C9-ALS (A), FUS-ALS (B), and TDP-ALS (C) motor neurons at days in vitro 25 compared with HC. The x-axis shows log2-fold change and y-axis -log10 (*P*-value). Proteins were considered significant based on a *P*-value cutoff of ≤ 0.1 and a fold change ≥ 1.3. Downregulated proteins are indicated in blue and upregulated proteins in red. Gene Ontology (GO) and Kyoto Encyclopedia of Genes and Genomes (KEGG) pathway analyses were performed to assess the biological functions of significantly altered proteins. ALS: Amyotrophic lateral sclerosis; HC: healthy control; iPSC: induced pluripotent stem cell.

To determine ALS mutation-specific proteomic signatures, protein expression in C9-ALS, FUS-ALS or TDP-ALS samples was compared to HC. Differentially expressed proteins were defined using a *P*-value ≤ 0.1 and a fold change ≥ 1.3, selected based on data distribution to ensure a balance between statistical significance and biological relevance. This analysis revealed 50 upregulated and 63 downregulated proteins in C9-ALS MNs (**Figure 2A**), 60 upregulated and 46 downregulated proteins in FUS-ALS MNs (**Figure 2B**), and 42 upregulated and 56 downregulated proteins in TDP-ALS MNs (**Figure 2C** and **Additional Table 3**). Gene Ontology (GO) enrichment analysis and KEGG pathway analysis were conducted to clarify the biological processes involved in each mutation genotype. For C9-ALS, GO analysis revealed upregulation of endoplasmic reticulum (ER) associated processes and nucleus import regulation were both up- and down regulated. KEGG pathways analysis confirmed the dysregulation of ER associated processes (**Figure 2A**). In FUS-ALS, several biological processes related to nerve growth and cytoplasmic microtubule organization were upregulated while metabolism-related processes were downregulated. Additionally, pathways related to ALS were upregulated, while several metabolic pathways were downregulated (**Figure 2B**). For TDP-ALS, upregulated GO terms included myelin assembly and mitochondrial translation, with downregulation in mRNA processing and splicing. KEGG pathway analysis related to the cytoskeleton was upregulated and spliceosome-related pathways were downregulated (**Figure 2C**).

**Additional Table 3:** ALS-mutation-specific alterations

(-log10Pvalue)	Fold change	Genes
**C9-ALS vs HC**		
1.273383205	-2.208932433	DCTN6
1.149930937	-0.621162913	PPP1R12A
1.737609243	-0.986196682	EIF3D
1.137605456	-1.0886968	CYB5B
2.064824009	-1.494809131	CHMP2A
1.006569686	-1.702702648	UGDH
2.389198845	-0.586621684	EDF1
2.106397885	-0.832352712	NDUFB4
1.787897076	-1.059929895	KRT18
1.150154394	-0.942087042	SLC3A2
1.279947031	-0.775869149	PCNA
1.983972439	-0.730525426	TPT1
1.106186946	-1.550366176	CD99
1.81292553	-1.461637199	GCSH
1.272455088	-1.771582861	DDX6
1.05337834	-0.69904016	LAP3
1.332536337	-0.850415203	PDIA3
1.590812738	-1.91617674	ALDH1B1
1.342446961	-1.066716408	DNAJA1
1.24281725	-1.338050287	RPS10
1.590047525	-0.625748375	CDC34
1.251118479	-0.679364425	CCT4
1.381748521	-2.334778493	ALDH5A1
1.031953029	-0.829437584	PGD
1.071382089	-0.874999546	ATP5MJ
1.121548534	-1.809717774	IGBP1
1.004933404	-0.776596234	AKR1C1
1.403056736	-0.69145903	SSBP1
1.76701833	-2.076578595	TFCP2
1.35400201	-0.973163263	LMAN2
1.523503753	-1.305800244	IDI1
1.180827853	-0.647253942	GANAB
1.446404203	-1.590687007	CRTC1
1.007642214	-0.750464356	CRACD
1.580010345	-2.412364446	NUFIP2
1.110228813	-1.138686197	CENPV
1.316256293	-1.616535395	GPSM1
1.026523668	-1.677337155	ARID3B
1.371210282	-0.685291758	CHERP
1.160521597	-3.528587987	PHC2
1.372481129	-1.233326139	H2BC26
1.224965906	-1.12411235	PLBD2
1.766626569	-0.584919263	GCN1
1.641772437	-1.326793372	FERMT2
1.031362987	-1.602474223	FAHD2A
1.041531921	-0.674038433	DDRGK1
1.157680781	-1.147071414	TMEM43
2.147372283	-1.022229396	TMEM109
1.381361416	-1.883011308	ILKAP
2.125042308	-2.462128758	EHMT1
1.119630144	-0.701653581	ENY2
1.192071511	-0.848135942	PDLIM7
1.073635406	-1.340244678	NANS
0.996791643	-2.018944514	STRN4
3.257669382	-1.10369218	MRPL17
1.602085456	-1.294427106	OLA1
1.415317161	-0.756341203	RAB21
1.583784459	-2.265835992	MAGED2
1.214281162	-1.580112138	COPS3
1.476214082	-1.172841476	SCAF8
1.370383036	-1.816546919	SUGT1
1.521808621	-2.332129026	PCDHGB6
1.457110917	-1.300023624	UTP18
2.383738964	1.304067646	ELAVL2
1.763490429	2.003905818	TPM1
1.754778627	1.152538076	PSMD12
1.604689598	1.812750735	PDHX
1.346407335	0.621679323	KPNA3
1.134906635	1.311560573	FABP7
1.216858828	1.526401142	STRN
1.001472119	0.914040791	CPNE3
1.043245631	1.530333705	PSIP1
1.025491738	0.644340415	LYPLA1
1.142101444	0.996100402	GLRX3
1.058151122	1.858424104	IPO7
1.362974783	1.443957208	TF
1.86459709	3.189189658	ITGAV
1.574585424	0.9543783	APRT
1.201956244	1.316446701	GNAI3
1.464557033	1.18682618	CLTA
1.578554382	0.746503899	CLTB
1.428794843	2.06833482	EIF2S2
1.089085007	1.300277084	SDHA
1.640051772	1.802652212	NUP153
1.138128268	1.564918695	BCAP31
1.098868302	0.64793738	SPCS3
1.219838787	1.101789131	SEC61A1
1.667687244	1.868764803	CDK5
1.310830482	1.134479469	GNA13
1.154023483	0.55397718	SPCS2
1.060184045	1.673709974	NSDHL
1.353306237	0.945585719	LSM12
1.629560499	1.578087223	EMC10
1.002058918	1.566691045	SUPT6H
1.074260831	1.830127541	ERMP1
1.063141108	1.100763504	C5orf24
1.211580326	2.399757743	ALDH16A1
1.225094579	1.494497379	AFAP1
1.297600311	1.291687347	PDCD6IP
1.554962651	2.341726199	CCT6B
1.448933653	0.987875741	NCSTN
1.060966161	2.313187405	MRPS31
1.1574096	0.709655273	UFD1
1.024626491	1.061880819	PHYHIPL
2.05315534	1.783526398	MTMR9
1.170760083	2.617953339	ARPC5L
1.259503365	1.428819901	PFDN4
1.107323605	0.750185784	TOMM22
1.683596974	2.759917339	ZCCHC3
1.400196932	0.959204584	KIDINS220
1.790440057	2.65687333	ZMYND8
1.375237286	0.683905399	CFL2
1.533578653	2.302512541	IER3IP1
**FUS-ALS vs HC**		
1.667429006	-3.716206964	PON2
1.3865979	-1.435559551	DVL2
1.216577508	-6.06224519	PLRG1
1.130678202	-2.306457743	GLRX3
1.151547063	-1.026616885	ACSL3
2.093331232	-2.396581863	LMNA
1.398007468	-1.127756834	PSAP
1.872995432	-0.916273869	PCNA
1.207556678	-1.816818595	MTHFD2
1.357350985	-1.737106484	GLB1
2.035134853	-1.047855353	NME2
1.601849559	-0.614456958	ECHS1
1.964708204	-0.599007746	PEBP1
1.600602334	-1.11594943	PPIF
1.399160941	-0.70228384	MCM5
1.126895603	-1.238016247	EIF2S3
1.05955017	-0.656320934	TMPO
1.834662644	-1.036516811	TUFM
2.050829151	-0.885487483	CDC34
2.316033493	-2.055138017	ALDH5A1
1.16679956	-0.950605004	HDGF
1.131617494	-1.07849836	HNRNPF
1.331370711	-1.850223964	BLVRA
1.414846841	-1.967006259	ATP5MJ
1.276541959	-0.582630629	RPS14
1.375541583	-1.181519911	RAP1A
1.036562649	-2.071688795	ABAT
1.121620391	-0.617084578	KGD4
1.016787016	-1.120581476	PTPN11
1.177052916	-0.905297187	CKAP4
1.168796291	-2.452709579	PPID
1.049807574	-0.981130035	LMAN2
1.222273571	-0.690204754	EFTUD2
1.100494294	-1.210613087	DPYSL2
1.124889703	-0.734628203	CENPV
1.077942574	-0.927347295	SCCPDH
1.191824211	-2.311102681	SCYL1
1.031319797	-1.089730075	CCT7
1.205833496	-1.32377999	PRMT1
1.301641343	-2.059216384	APOO
1.075078148	-0.82438404	TMEM109
1.118964722	-0.617460612	NANS
1.164768312	-4.327904522	HIF1AN
1.055034236	-1.084928428	TRMT112
1.447617056	-0.969663726	RAB21
0.998934006	-1.870793906	WNK2
1.705643673	3.120320767	NELFB
1.006921571	0.836503714	MAPT
1.06422224	1.889766621	NCAM1
2.310151994	1.135139157	TUBB6
1.442310552	1.543391613	CBX4
1.437651301	1.628438404	PDHX
1.101714842	0.865122198	ABLIM1
1.082925829	1.99853645	ANKRD17
1.160632048	1.495946216	PSIP1
1.502361189	0.677499772	CLTB
1.682136731	0.559287092	HARS1
1.528186974	0.99525984	SCG2
1 104043498	1.794331967	SMPD1
1.231072935	1.223496861	XRCC1
1.253010659	1.768922769	EIF2S2
1.061959183	1.111163183	NMT1
1.043848859	0.613640193	CORO1A
1.461385791	0.961517071	ACTR1B
1.349769866	2.142880877	RBM25
1.424855761	2.37710519	NUP153
1.918407084	2.426560807	LIG3
1.009283266	1.30453781	IST1
1.058539439	1.662636213	UFM1
0.993114793	0.772947621	UBXN1
1.455958384	2.058081699	MPP2
1.434944534	0.648136898	MAPRE2
1.087776806	1.66935467	NSDHL
1.126938047	1.377122379	ADRM1
1.153285587	1.831245904	MRPL23
1.567460486	2.065047205	CCDC88A
1.027386639	1.027834691	VPS26B
1.076583108	1.89018512	LBH
1.234382001	1.849151115	UBAP2
1.056747445	3.816873208	FIP1L1
2.080986002	1.112639304	CRTC1
1.104910557	1.62889272	MPRIP
1.237238167	1.29884236	KYAT3
1.048315773	1.684999069	EIF3M
1.018845006	1.583056405	ERMP1
1.408796667	1.116170051	C5orf24
1.004513837	1.807706959	PHACTR4
1.031846231	1.814456557	AFAP1
0.993333826	1.441153476	AMER2
1.047973352	2.586161499	ATAD1
1.476741617	1.121945603	MICAL1
1.00699337	2.008667797	SNRNP27
1.663908231	1.839863101	CCT6B
1.291141831	4.102660393	CCDC43
1.04677544	1.651903235	PPP1R9B
0.991721831	1.535820703	KATNAL1
1.11623258	2.315263137	QTRT1
1.449705738	1.567056353	TOLLIP
1.021131136	1.602614919	PPCS
1.157920834	1.496504841	PFDN4
0.991020458	3.224820361	ZCCHC3
1.468743262	1.483994635	STAU2
1.02398659	0.739069101	KIDINS220
1.028614951	1.999022961	ZMYND8
1.183153205	1.022226725	COA3
1.13966033	1.975059047	NDUFB9
**TDP-ALS vs HC**		
1.478170113	-0.859907271	NCAM1
1.748073867	-3.647117327	RTL8C
1.119410406	-0.666631905	PGRMC2
2.40109805	-1.580936968	ALDH1A2
1.914079453	-2.117036412	ASMTL
1.124404182	-0.790185483	AIFM1
1.063734057	-2.256386787	COL1A2
1.115359116	-3.359109189	SNRPB2
2.204459487	-2.444611892	LAMC1
1.013766168	-0.753272538	ACADM
1.157115191	-0.91396899	ETFA
1.05796675	-1.17571414	UBTF
1.813759942	-1.051540693	POLR2A
0.997293636	-1.714873058	ERP29
1.560704052	-1.13396848	PPIF
1.478459533	-1.885619809	CDKN1A
1.482251524	-0.804295673	ECI1
1.354954554	-2.158105349	PSEN1
1.204882635	-1.145503547	ALDH5A1
1.100656252	-1.429714388	TPD52
1.127237012	-2.270047784	RRP1
1.062790726	-0.559866885	UBE2D3
1.274704217	-1.486298362	COPS2
1.173783278	-1.850421198	RAB11A;RAB11B
1.138068955	-2.233933916	EFNB1
1.023511014	-2.246357473	CDK6
1.182418116	-0.928531881	AKR1C1
1.005685786	-0.639313275	GRSF1
2.265011709	-0.5783201	SF3A3
1.21707366	-1.289880837	GFUS
1.512934987	-0.66290586	IDI1
1.038070569	-3.527462842	RAB35
1.038079988	-1.877125442	DGUOK
1.989521653	-1.539478615	HSDL2
1.48453905	-1.996229634	TRMT10C
1.237932775	-0.738725583	CYFIP1
1.093299894	-1.147802238	POGZ
1.109580426	-0.710353533	SCD5
1.687004723	-1.846884021	SRRM1
2.353288564	-1.4139135	BRSK1
1.235791831	-2.217116753	CTNNBL1
1.01438894	-0.583220875	SYMPK
1.261128225	-1.840385407	SCLY
1.123208778	-1.518115905	CCT7
1.469800006	-1.055873285	NIPSNAP1
1.545584032	-1.837763222	MRPL45
1.080797846	-0.893552995	GRPEL1
1.078845663	-1.770014062	NCOA5
2.241091821	-1.910784841	HIF1AN
1.319594846	-1.265293616	ADPRS
1.153132764	-1.522696009	EIF3K
1.117039436	-1.067524949	SRP68
1.01841683	-0.975847974	RALY
1.003064966	-1.670849378	CORO1C
1.334551098	-1.731381853	PA2G4
1.092088955	-1.87902163	RTCB
1.770759858	2.233638366	ACTB
1.161795101	1.099624674	TPM1
1.341680902	0.733230333	CLIC1
1.116318174	4.334071343	ZNF185
1.000103157	0.784587413	VGF
1.044232323	0.95192893	FABP7
0.997326802	3.062469816	EPB41L2
1.346817026	1.875491869	SEC24A
1.902360719	1.228127527	TF
3.200546128	3.985173464	ITGAV
1.209456807	1.366835101	POLR1D
1.915385337	0.678631163	SCG2
1.000814687	0.587746988	EZR
1.103551583	1.263788611	XRCC1
1.712327399	0.917797965	MYH9
1.159827124	1.212959091	CNTNAP1
1.105445009	1.217694658	GSTO1
1.105439236	1.284852478	SLC7A5
1.458704087	1.085869839	CRYZ
1.039148273	0.743179691	MLLT11
0.996243135	1.435193717	CSRP2
1.507109661	2.183273425	MRPL23
2.113350299	2.146666549	UBAP2
1.295716145	1.472358278	SLC25A24
1.072954289	0.841244364	GIGYF2
1.345559918	1.482895957	PHLDB1
1.285214728	2.321538138	FTSJ3
1.183431953	1.113050604	AFAP1
1.549279108	2.649439722	GADD45GIP1
1.179602462	2.140027374	PDLIM5
1.139277324	3.427910556	CCDC43
1.032660756	1.292992216	EIF2A
1.440146706	1.853337163	FOXP1
1.4317921	5.877726376	GLIPR2
1.200765086	0.842688771	UPF2
1.046601034	0.900428355	MKKS
1.129629712	0.622687741	ARL8B
1.195410887	0.898061009	AKAP8L
1.890283197	1.154209488	SNX6
1.576508036	1.657705734	EPB41L3
1.120775101	0.76632853	LSM2
1.110805904	2.773499966	MRPS23

**Figure 3:**
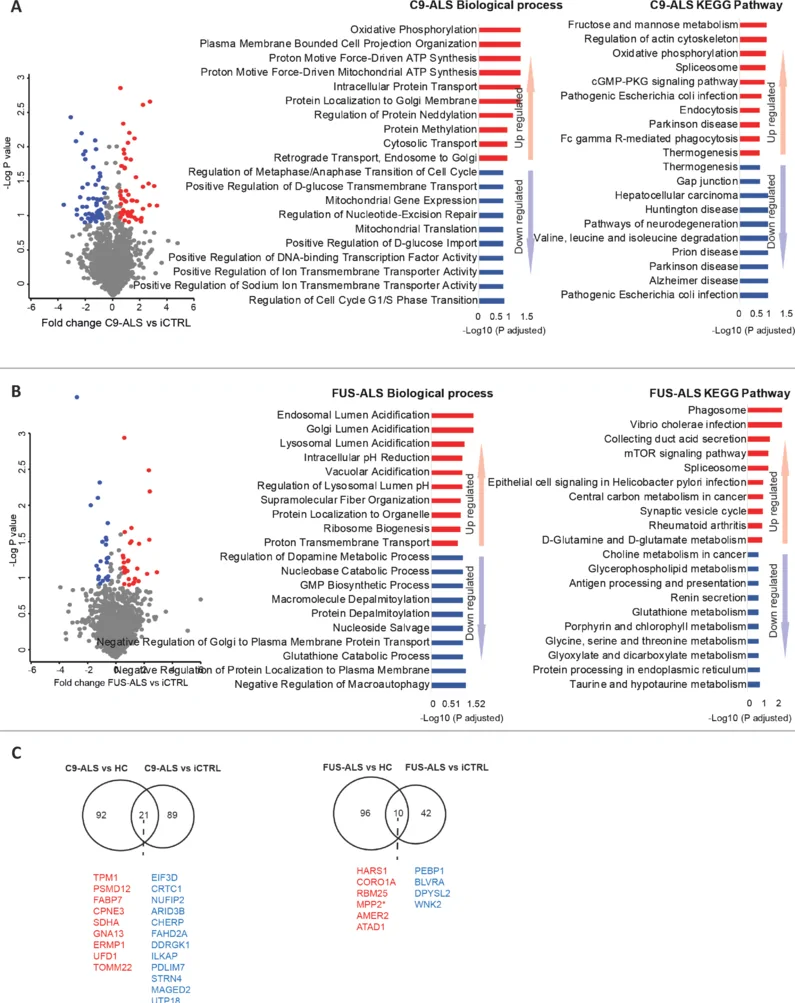
Proteomic changes in iPSC-derived motor neurons caused by C9ORF72 or FUS mutations. (A) Volcano plot showing differentially expressed proteins in C9-ALS MNs compared to their isogenic controls (iCTRL) lacking the repeat expansion. Gene Ontology (GO) and Kyoto Encyclopedia of Genes and Genomes (KEGG) pathway analysis was performed to assess the biological functions of significantly altered proteins. (B) Volcano plot showing differentially expressed proteins in FUS-ALS MNs relative to their iCTRL. GO and KEGG pathway analysis were performed for significantly altered proteins (*P* ≤ 0.1, fold change ≥ 1.3). (C) Venn diagram illustrating shared dysregulated proteins in C9-ALS and FUS-ALS MNs, compared to HC or matching iCTRL. Downregulated proteins are indicated in blue and upregulated proteins in red. Asterisks indicate proteins regulated in the opposite direction in FUS-ALS versus iCTRL. ALS: Amyotrophic lateral sclerosis; HC: healthy control; iPSC: induced pluripotent stem cell; MN: motor neurons.

To further assess ALS mutation-specific changes, we compared the C9-ALS and FUS-ALS proteomes to that of iCTRL, which was generated from these lines. This analysis revealed 54 upregulated and 57 downregulated proteins in C9-ALS and 30 upregulated and 22 downregulated proteins in FUS-ALS (**Figure 3A** and **B**). For C9-ALS, GO analysis revealed upregulation of oxidative phosphorylation and (mitochondrial) ATP synthesis, while cell cycle-related processes were downregulated. In FUS-ALS, biological processes associated with endo-lysosomal organization were upregulated, whereas ER organization and macroautophagy were downregulated. Interestingly, several proteins were significantly altered in comparison to both HC or iCTRL samples (**Figure 3C**). Proteins exclusively altered in C9-ALS were involved in Golgi vesicle transport, protein localization to ER, and oxidative stress. In FUS-ALS, such differentially expressed proteins were associated with intermediate filament bundle assembly. Overall, this analysis provides valuable insights into the molecular mechanisms underlying ALS related to *C9ORF72*, *FUS,* and *TDP-43* mutations, highlighting potential pathways for targeted therapeutic strategies.

### Common proteomic changes in amyotrophic lateral sclerosis motor neurons

**Figure 4:**
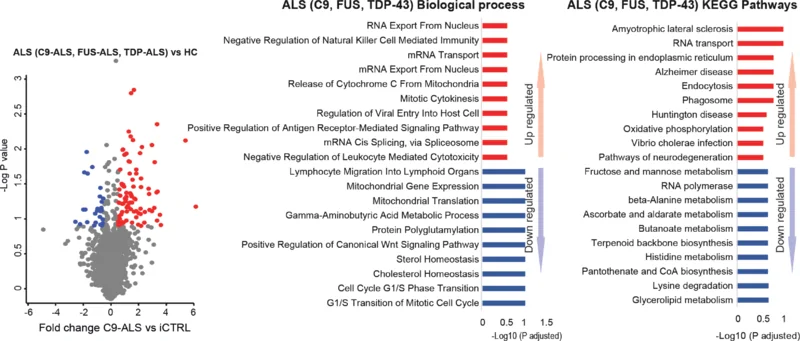
Common proteomic changes in ALS iPSC-derived motor neurons. Volcano plot demonstrating differentially regulated proteins in ALS (C9-ALS, FUS-ALS, and TDP-ALS) compared to HC motor neurons. The x-axis shows log2-fold change and the y-axis the -log10 (*P*-value). Proteins with a *P*-value cut-off of ≤ 0.1 and a fold change ≥ 1.3 were considered significant. Downregulated proteins are shown in blue and upregulated proteins in red. Gene Ontology (GO) and Kyoto Encyclopedia of Genes and Genomes (KEGG) pathway analysis was performed for the significant proteins. ALS: Amyotrophic lateral sclerosis; HC: healthy control; iPSC: induced pluripotent stem cell.

Following the characterization of mutation-specific proteome signatures in ALS, we sought to identify common proteomic alterations across these mutations. To achieve this, we combined data from C9-ALS, FUS-ALS, and TDP-ALS MNs and performed comparative analyses against HC samples. Proteins were considered significantly regulated based on a threshold of *P*-value ≤ 0.1 and a fold change ≥ 1.3. This integrative analysis identified 89 upregulated proteins and 26 downregulated proteins (**Figure 4** and **Additional Table 4**). GO analysis revealed upregulation of biological processes related to mRNA and RNA transport, while cell cycle-related processes were downregulated. Additionally, KEGG pathway analysis highlighted the upregulation of pathways involved in ALS, RNA transport, and ER-related pathways, whereas pathways related to metabolism were downregulated. Notably, NEFL (neurofilament light chain), a critical biomarker in ALS pathology, was significantly upregulated in all conditions. Several of these dysregulated processes were also observed in some of the mutation-specific analyses. In conclusion, our integrative proteomic analysis highlights key shared molecular signatures across distinct ALS-associated mutations, offering insights into common pathogenic mechanisms at the protein level.

**Additional Table 4:** ALS general alterations

(-log10Pvalue)	Fold change	Genes
**Sign down regul. in ALS vs HC**		
1.788792901	-1.420988083	GMDS
1.208257364	-0.710558186	PSAP
1.394173062	-0.834662027	POLR2A
1.510946921	-0.819920746	PPIF
1.163446189	-1.220193377	ALDH1B1
1.363589542	-0.592358871	CDC34
1.993702959	-1.866139819	ALDH5A1
1.201508615	-1.051009077	ATP5MJ
1.214732761	-2.13803529	CDK6
1.320375527	-0.712859252	AKR1C1
1.226873164	-1.042135097	TFCP2
1.015649441	-0.79573263	LMAN2
1.045866133	-1.823093946	LSAMP
1.237655569	-0.738794974	IDI1
1.004122189	-1.293003662	FAM50A
1.194610373	-0.816412757	HSDL2
1.019796147	-2.384805502	PHC2
1.116455493	-0.840445153	AGPAT1
1.028167193	-0.640947736	NIPSNAP1
1.038878939	-1.19062485	MRPL45
1.085075452	-0.615707525	WNK1
1.699498363	-1.784621298	EHMT1
1.051107038	-2.756032626	MRPS7
1.718659698	-2.058587818	WNK2
1.219554412	-1.967676931	PCDHGB6
1.002402188	-0.687035628	CFAP20
**Sign up regul. in ALS vs HC**		
1.245119569	1.260716642	MYEF2
1.29134593	0.95611488	ELAVL2
1.194609669	1.975865045	EIF4G3
0.997958856	2.335769039	RUFY3
1.004938228	3.883286675	SHF
1.757975045	0.730510569	TUBB6
1.304814445	0.660277197	PSMD12
1.29302867	0.88511664	CBX4
2.78491871	1.589081465	PDHX
1.194833575	3.208375168	ZNF185
1.957267292	1.067294006	FABP7
1.386559773	1.222661378	SART1
1.157606179	2.321075996	EPB41L2
1.36325035	1.822959726	GSTZ1
1.555105105	0.968599319	SNX3
1.070581746	3.592197048	GGCT
2.270995584	1.412722284	PSIP1
1.092432919	0.711231744	GLS
1.253277599	6.602625385	FKBP9
1.368407857	1.092201254	IPO7
1.307042442	1.448769805	SEC24A
1.312426627	1.496772408	STAMBP
1.426473737	2.964391559	ITGAV
1.549052602	0.883164347	CLTA
1.008253807	0.592287138	COX6C
1.525304955	1.752610731	UMPS
1.440239063	1.211123123	SMPD1
2.151328305	1.717963109	EIF2S2
1.042111422	0.974529585	DNAJB1
1.404183638	0.749462021	MYH10
1.041654251	0.635868369	TALDO1
1.049815992	0.575143182	CAPZA2
1.619117997	1.673134111	NUP153
1.24107116	3.387759134	PLCD1
1.973784958	1.208828702	BCAP31
1.220767024	1.550216436	BID
1.041558173	2.604562849	ATP5F1E;ATP5F1EP2
1.015504935	0.739598734	SEC61A1
1.190649212	1.780542228	RHOB
1.1285296	0.659642021	GRB2
1.291327689	0.870270861	GSTO1
1.183203239	2.631279286	NUCB2
2.020036594	0.911687181	CRYZ
1.757538483	1.97016394	MPP2
1.188244911	1.255887702	NSDHL
1.151422115	0.825266802	ATP6V1F
1.043039589	0.946368218	NCBP3
1.436774187	1.516688628	UBAP2
1.010324955	2.126039697	SPECC1L
1.417104507	1.559824568	MPRIP
1.524317847	1.026680798	KYAT3
1.461036477	3.618108327	RAPH1
2.202125234	1.535122409	ERMP1
1.635169595	2.338838024	FTSJ3
1.522360108	2.333268713	TMEM123
1.867193966	1.408374075	AFAP1
1.211310191	3.000473082	PCYOX1L
1.975385834	2.277566723	ATAD1
1.700312616	1.976565182	SREK1
2.829245687	1.788654886	CCT6B
1.308437247	0.613619501	NCSTN
1.113245651	1.992368452	MRPS31
1.048455934	0.921508573	NECTIN2
1.393548093	1.603215933	SCAMP4
1.266991898	1.324662359	LRRC59
1.025139012	0.819041236	OPTN
1.499539812	2.942380443	THOC1
1.310966346	1.64511562	PDLIM5
2.36985307	3.600011017	CCDC43
1.251783798	1.346951106	MTMR9
1.861705046	1.477976967	PPP1R9B
1.355467368	1.921285646	QTRT1
1.352192107	1.184033651	EIF2A
1.169530074	2.175796354	CSTF2T
1.700037018	1.453777478	FOXP1
2.149780621	5.78064545	GLIPR2
1.144428321	3.80502789	GORASP2
1.562183263	0.827076639	UPF2
2.087203044	3.20580353	ZCCHC3
1.448283256	3.560858458	TEX10
1.228131948	2.307627022	KCMF1
1.182270304	0.89298934	NUDT5
1.484952293	0.664887334	KIDINS220
2.06271116	2.407542109	ZMYND8
1.066597614	1.220694677	KMT2B
2.027031991	0.938284083	SNX6
1.325799085	1.132035217	EPB41L3
1.03869895	1.666640155	CIAO2B
1.858696346	3.41485865	MRPS23

### Mesenchymal stem cell–derived extracellular vesicles can reverse amyotrophic lateral sclerosis phenotypes *in vitro*

Differential protein expression patterns can be exploited to dissect disease pathways and identify therapeutic targets. In addition, proteomic signatures can be used to assess the effect of therapeutic interventions at the protein/pathway level. Although several studies have reported beneficial effects of MSCs in ALS rodent models (Frawley et al., 2024; Shi et al., 2025), their effect in human *in vitro* models of this disease is unknown. Further, the therapeutic effects of these cells are likely primarily mediated through their secretome. For example, by proteins present in EVs released by MSCs (Varderidou-Minasian and Lorenowicz, 2020). Interestingly, MSC-EVs are known to influence neurite growth and branching (Van Hoecke et al., 2025) and several ALS genetic defects cause altered neurite growth *in vitro*. For example, cultured FUS-ALS MNs display shorter neurites (Stoklund Dittlau et al., 2021). Therefore, to establish whether MSC-EVs can reverse ALS-associated cellular phenotypes, their effect on a neurite growth defect observed in FUS-ALS MN cultures was tested.

**Figure 5:**
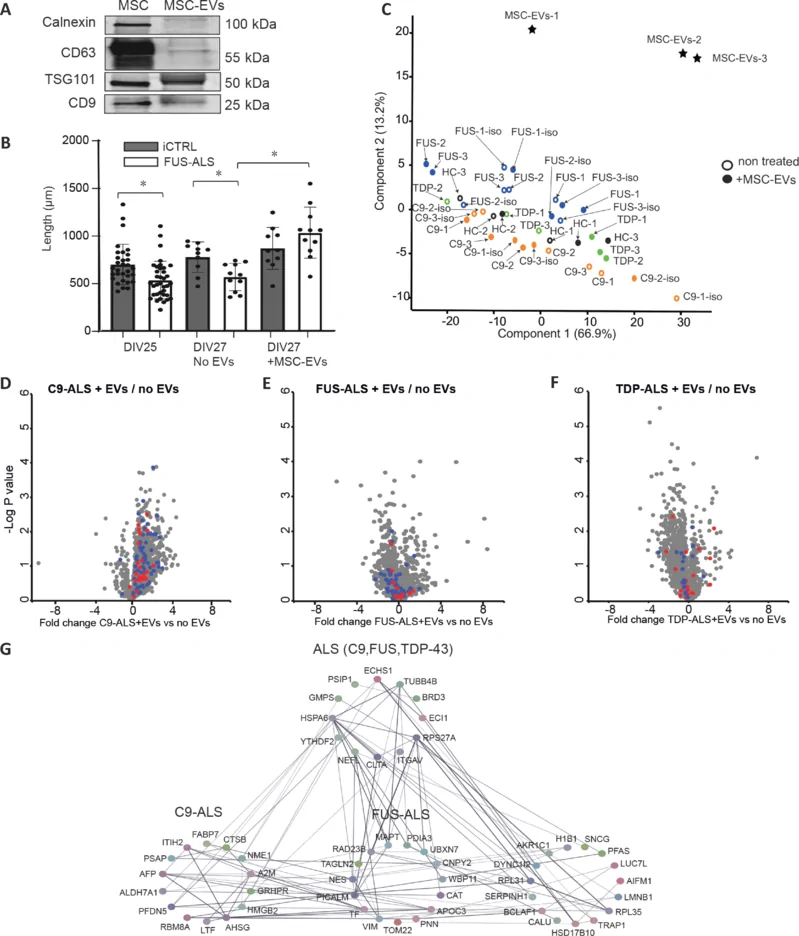
Treatment of iPSC-derived motor neurons with MSC-EVs. (A) Western blot analysis detected the exosomal markers CD63, CD9, and TSG101 in cell lysate (MSC) and MSC-EVs, while the absence of the endoplasmic reticulum protein Calnexin confirmed minimal contamination by non-EV material. (B) Quantitative analysis of neurite length in FUS-ALS and iCTRL motor neurons (MNs) prior to and following two days of MSC-EV treatment. Data represent the mean ± SEM from three independent FUS-ALS lines (FUS-1, FUS-2, and FUS-3) and their respective isogenic controls (FUS-1-iso, FUS-2-iso, and FUS-3-iso) (*n* = 3). *P < 0.05. Statistical significance was determined using an unpaired two-tailed Student’s *t*-test. (C) Principal component analysis of the proteomes of MSC-EVs (star), non-treated iPSC-derived MN samples (open circle), and MSC-EV-treated (closed circle) MN samples. Samples include ALS lines, healthy control (HC), and iCTRL. (D–F) Volcano plot showing differentially expressed proteins in C9-ALS, FUS-ALS and TDP-ALS MNs treated with MSC-EVs compared to untreated MNs. Significantly regulated proteins in C9-ALS relative to HC are highlighted in red (upregulated) and blue (downregulated). (G) Protein–protein interaction networks of the most significantly altered proteins following MSC-EV treatment are shown for each mutation individually, as well as for all ALS mutations combined. ALS: Amyotrophic lateral sclerosis; HC: healthy control; iCTRL: isogenic (iso) control; iPSC: induced pluripotent stem cell; MNs: motor neurons; MSC-EVs: mesenchymal stem cell-derived extracellular vesicles.

MSCs were derived from bone marrow aspirated from third-party healthy donor, approved by the Dutch Central Committee on Research Involving Human Subjects (CCMO, Biobanking for MSC, NL41015.041.12) and EVs were isolated using differential ultracentrifugation steps (Vonk et al., 2018). Western blot analysis confirmed expression of the exosomal markers CD9 and CD63, TSG101, and absence of Calnexin, an integral protein of the ER (**Figure 5A**). The detection of CD9 and CD63 was enhanced by incorporating an additional sucrose-gradient enrichment step (**Additional Figure 4A**). Nanosight particle tracking analysis indicated that the nominal size of MSC-EVs was approximately 142 nm with a concentration of 6.4 × 10^6^ particles per milliliter (**Additional Figure 4B**). To assess the effect of MSC-EVs on the reduced neurite growth displayed by FUS-ALS MNs, FUS-ALS and iCTRL cultures (FUS-3 and FUS-3-iso) were exposed to MSC-EVs (EVs derived from 4 × 10^6^ MSCs per well of a 24-well plate), in line with previous data showing therapeutic efficacy (Vonk et al., 2018). On both days *in vitro* DIV25 (prior treatment) and DIV27 (after 2 days of treatment), FUS-ALS MN cultures displayed reduced neurite length as compared to iCTRL in the absence of MSC-EV treatment (**Figure 5B**). Two-day treatment with MSC-EVs did not significantly alter neurite growth in iCTRL conditions but reversed the neurite length reduction observed in FUS-ALS MN cultures. Together, these experiments show that MSC-EVs can reverse ALS-associated phenotypes in human *in vitro* MN models.

To begin to understand how MSC-EVs may affect ALS iPSC-derived MNs at the protein level, lysates from three independent MSC-EV isolations (100 µg) were subjected to mass spectrometry. A total of 1967 proteins were identified with 1% FDR, including the exosomal markers CD63, CD81, and CD9 (Calnexin was not detected). GO analysis of the MSC-EV proteome revealed enrichment in biological processes related to cellular localization, vesicle-mediated transport, and exocytosis (**Additional Figure 4C**). The PCA plot further revealed a distinct protein composition in MSC-EVs compared to MNs (**Figure 5C**). Specifically, the MSC-EV proteome was enriched for defense immunity and cytoskeletal protein classes, while it contained fewer gene-specific transcriptional regulators (**Additional Figure 4D**). Additionally, storage proteins such as ferritin were identified in MSC-EVs but absent in neuronal samples, suggesting differences in metabolic requirements between MSCs and neuronal cells. The top 25 most abundant MSC-EV proteins included albumin, thrombospondin-1, alpha-2-macroglobulin, fibronectin 1, and complement C3, which are known to be involved in immune modulation, extracellular matrix remodeling, and tissue repair (**Additional Figure 4E**).

### Proteomic signature after mesenchymal stem cell–derived extracellular vesicle treatment of motor neurons carrying specific amyotrophic lateral sclerosis mutations

To explore the molecular mechanisms underlying the rescue of neurite growth phenotypes observed in FUS-ALS MNs by MSC-EVs, we performed proteomic analysis of MNs to examine changes in protein expression induced by MSC-EV treatment in MNs carrying three different ALS mutations. We focused on the proteins that were significantly up- or downregulated in ALS samples from each mutation (C9-ALS, FUS-ALS, and TDP-ALS) and visualized these changes in volcano plots (**Figure 5D–F**). Interestingly, levels of most significantly dysregulated proteins in ALS were either normalized or shifted toward the opposite direction after MSC-EV treatment. This suggests that MSC-EVs may help restore normal cellular function by modifying the expression of key proteins involved in ALS pathogenesis. This was confirmed by analysis of the top 10 most significantly up- and downregulated proteins for each mutation compared to HC and their normalized expression following MSC-EV treatment. For C9-ALS MNs, MSC-EV treatment predominantly affected DNA binding, fatty acid transport, and mRNA splicing. For FUS-ALS, treatment had a major impact on endocytosis, apoptosis and protein transport. For TDP-ALS, MSC-EV exposure targeted metabolic processes, and apoptotic pathways (**Additional Figure 5A–C**).

Next, common proteomic changes in ALS MNs were assessed following MSC-EV treatment (**Additional Figure 5D** and **E**). As observed in mutation-specific analyses, treatment with MSC-EVs led to the normalization of the proteomic profile in ALS cells. Specifically, MSC-EV treatment targeted several key biological processes that were dysregulated across all ALS mutations, including oxidation, mRNA splicing, and autophagy. These processes are commonly disrupted in ALS pathogenesis, and their restoration by MSC-EV treatment suggests a broad therapeutic potential for MSC-EVs in mitigating the molecular abnormalities shared across different ALS mutations. We further highlighted the proteins showing the most pronounced expression changes following MSC-EV treatment, both for each individual mutation and across all mutations combined, using protein–protein interaction network analysis (**Figure 5G**).

To further validate the anti-apoptotic effects of MSC-EVs, we treated both HC and C9-ALS MNs with MSC-EVs for 2 days and measured the levels of cleaved Casp-3, a key marker of apoptosis. The results revealed a significant increase in Casp-3 levels in C9-ALS MNs, indicating elevated apoptotic activity. Notably, MSC-EVs treatment led to a marked reduction in Casp-3 levels, suggesting that MSC-EVs effectively mitigate apoptosis in C9-ALS MNs. These findings provide additional support for the potential of MSC-EVs as a therapeutic strategy to protect MNs in ALS (**Additional Figure 6**).

## Discussion

iPSC-derived MNs are widely used to study ALS; however, our understanding of the disease-associated proteomic changes in these models remains incomplete. These are, however, critical as they could serve as starting points for therapy development. Here, we conducted a comparative proteomic analysis of iPSC-derived MNs carrying ALS-linked mutations in *C9ORF72*, *TARDBP*, or *FUS*. This revealed both mutation-specific and shared proteomic signatures, unveiling common and divergent disease mechanisms. Furthermore, it allowed us to show the ability of MSC-EVs to reverse ALS-associated proteomic changes in MNs carrying different gene mutations and restore functional defects in FUS-ALS neurons specifically. These findings unveil key proteomic signatures underlying ALS and identify MSC-EVs as a potential therapeutic approach for this disease.

### Mutation-specific and shared disease pathways in amyotrophic lateral sclerosis motor neurons

Proteomic analysis of iPSC-derived MNs identified novel as well as previously reported mutation-specific changes. Several of the altered proteins belonged to pathways previously implicated in ALS, confirming the validity of our approach. In C9-ALS-specific MNs, we observed dysregulation of ER-Golgi vesicle transport and oxidative phosphorylation. Disrupted ER-Golgi vesicle transport was previously reported in C9-ALS spinal cord tissues and iPSC-derived MNs (Sultana et al., 2024). A reported role of C9ORF72 is to regulate membrane trafficking events, including ER-Golgi transport (Tang et al., 2020). Impaired ER-Golgi trafficking can disrupt the localization of proteins to the ER, leading to the accumulation of misfolded proteins, induction of ER stress, and activation of apoptotic pathways, all of which are upregulated in our C9-ALS MNs. Toxic dipeptide repeat proteins produced from the C9ORF72 expansion may further contribute to these trafficking impairments and stress responses. Changes associated with FUS-ALS included intermediate filament bundle assembly. Abnormal assembly of intermediate filaments has been previously shown in the formation of axonal spheroids, focal swellings characterized by accumulation of neurofilaments, which are thought to disrupt axonal transport and contribute to neuronal dysfunction (Neumann et al., 2009). The disruption of axonal transport may underlie some of the morphological abnormalities frequently observed in FUS-ALS MNs. Interestingly, studies examining neurite architecture in FUS-ALS models have reported conflicting findings, with some showing increased axon branching and neurite complexity, while others report reduced neurite length and simplified arborization (Garone et al., 2021; Hawkins et al., 2022). These variations may reflect differences in disease stage, cellular context, or compensatory mechanisms. It is possible that early-stage FUS pathology leads to hyperbranching as a compensatory response to impaired transport, while progressive accumulation of cytoskeletal abnormalities eventually results in neurite shortening and degeneration. Finally, TDP-ALS proteomic changes were linked to mitochondrial translation and RNA splicing. TDP-43 cytoplasmic aggregates were shown to localize to mitochondria and disrupt several respiratory chain complex proteins, resulting in energy deficiency and MN loss (Lépine et al., 2022). Furthermore, the loss of nuclear TDP-43 resulted in increased inclusion of cryptic exons, leading to frameshifts and mRNA substrates for nonsense-mediated decay, a major pathway that regulates mRNA stability (Mehta et al., 2023). These mutation-specific processes may be explained due to altered protein or RNA interactions. Previous interactome studies have shown that indeed, C9ORF72, TDP-43, or FUS display distinct protein and RNA interacting partners, which likely impair a range of distinct pathways underlying ALS pathology (Cheng et al., 2025; Yap et al., 2025). 

Our data also show that mutations in these ALS-associated genes can trigger several common cellular pathways, such as RNA transport and mitochondrial translation. In this study, we also report novel mechanisms that may contribute to ALS pathology, including the downregulation of fatty acid degradation, a topic that remains largely unexplored in ALS research. Notably, recent findings in C9-ALS also highlight this pathway as disrupted (Giblin et al., 2025). Given their high metabolic demands, MN rely heavily on mitochondrial β-oxidation of fatty acids to produce acetyl-CoA, which enters the TCA cycle to drive ATP synthesis. Impairment of this process may result in energy deficits that render MNs particularly vulnerable to cellular stress and degeneration. This mechanistic insight aligns with epidemiological evidence showing that higher dietary intake of fatty acids is associated with reduced ALS risk and improved survival after disease onset, supporting a potentially protective role for lipid metabolism in ALS progression (Giblin et al., 2025).

Several interesting candidates were identified among the top ten most significantly up- and downregulated proteins shared across the three ALS mutations. This included NEFL, which marks disrupted cytoskeletal integrity. NEFL accumulation is a recognized pathological hallmark of ALS and has been validated as a fluid biomarker for disease progression (Wong et al., 2000; Witzel et al., 2024). This strengthens the biological relevance of our proteomic findings and underscores the link between cytoskeletal dysfunction and MN degeneration in ALS. Moreover, its dual role as both a pathological marker and a quantifiable biomarker makes NEFL particularly valuable for monitoring disease activity and evaluating therapeutic responses in future studies. Together, our results indicate that the protein changes can serve as a valuable starting point to better understand ALS pathogenesis and ultimately for the design of novel treatments.

### Therapeutic effect of mesenchymal stem cell–derived extracellular vesicles

Our data reveal several dysregulated cellular pathways, each likely contributing to the multifactorial pathogenesis of ALS. While most current experimental therapies target individual genes or pathways, our findings support a broader, multimodal therapeutic strategy. We demonstrate that MSC-EVs can have therapeutic effects in ALS MN cultures. Previous studies have shown that MSC-EVs improve motor performance in ALS mouse models (Gschwendtberger et al., 2023; Turano et al., 2023). However, the underlying molecular mechanisms remained largely unknown. We exploited our proteomics data and approach to investigate the effect of MSC-EVs on iPSC-derived MNs from ALS patients with diverse genetic backgrounds. Our analysis revealed that MSC-EVs carry heterogeneous cargo, including enzymes, immune-related proteins, and transcriptional regulators, enabling them to simultaneously modulate multiple cellular pathways. This multimodal effect was supported by our proteomics data, which showed that MSC-EV treatment reversed both mutation-specific and commonly dysregulated protein changes in ALS MNs. For example, in FUS-ALS MNs, MSC-EV treatment normalized proteins associated with endocytic trafficking and apoptosis—key processes involved in cytoskeletal stability, axonal transport, and neuronal survival—which may underlie the observed rescue of neurite outgrowth defects. In C9-ALS MNs, MSC-EVs restored protein levels related to ER-to-Golgi vesicle transport, while in TDP-ALS MNs, MSC-EVs modulated proteins linked to metabolic processes and apoptosis. These findings are consistent with prior studies reporting that MSC-EVs exert neuroprotective effects through modulation of apoptosis, inflammation, and axonal growth (Giunti et al., 2021; Wang et al., 2024; Poongodi et al., 2025). Importantly, we also treated HC and iCTRL MNs with MSC-EVs and did not observe a significant overall increase in the expression of the proteins that were altered in ALS MNs. This suggests that the uptake of MSC-EV cargo by healthy neurons does not result in a harmful accumulation of proteins. One possible explanation is that healthy cells possess efficient proteostasis and clearance mechanisms, such as the ubiquitin-proteasome system and autophagy, which allow them to selectively degrade or recycle excess proteins delivered by MSC-EVs. In contrast, ALS MNs, which are already under proteostatic stress, may benefit more from the exogenous support provided by MSC-EV proteins. These subtype-specific responses to MSC-EVs highlight the adaptability of MSC-EVs and underscore their potential as a precision-based, multimodal therapeutic agent capable of accommodating the heterogeneity of ALS. Beyond direct molecular effects, MSC-EVs may also exert indirect influences. For example, MSC-EVs may modulate the MN secretome, thereby altering the extracellular environment to promote the survival and function of neighboring MNs. Translation of MSC-EV therapy to ALS clinical application will require addressing several key challenges, including the standardization of EV production and dosing protocols, a deeper mechanistic understanding of their active components, the development of effective delivery strategies to the central nervous system, and the evaluation of long-term safety. Incorporation of MSC-EVs into combinatorial treatment strategies may enhance their impact and offer new avenues for disease modification.

This study has some limitations that should be noted. First, although we minimized and controlled for batch-to-batch variation, the experiments were ultimately conducted using only two independent differentiation batches. Additional batches will be required to further strengthen the reproducibility and robustness of the findings. Second, the MSC-EVs used in this study were obtained from a single donor. Therefore, the observed regenerative effects on neurite outgrowth and the anti-apoptotic responses may reflect donor-specific properties. Future studies including MSCs from multiple donors will be necessary to evaluate the consistency and generalizability of these effects.

In conclusion, our data reveal both mutation-specific and shared proteomic signatures linked to different ALS mutations. Some of these findings confirm pathways previously implicated in ALS, while others provide a valuable framework for dissecting novel disease mechanisms. In addition to being exploited for dissecting disease pathways or for therapy development, differential protein expression patterns can be used to assess the effect of therapeutic interventions at the protein/pathway level. In line with this, we show the ability of MSC-EVs to reverse proteomic changes in MNs with different ALS genetic backgrounds, in addition to being able to rescue axonal deficits in FUS-ALS. These findings highlight key molecular pathways in ALS at the protein level and support the potential of MSC-EVs as a possible therapeutic approach in ALS.

## Additional files:

***Additional Figure 1:*** *Characterization of iPSC-derived motor neuron cultures.*

Additional Figure 1 Characterization of iPSC-derived motor neuron cultures.
(A) Representative images of iPSC-derived MNs from different control and ALS iPSC lines immunostained for iPSC markers (NANOG) and MN markers (ISL1, TuJ, HB9, CHAT, and SMI32) 24 days in vitro. Dapi is in blue. Scale bars: 250 μm. (B) Cell viability of MNs with different genetic backgrounds. Each data point represents an independent differentiation. ALS: Amyotrophic lateral sclerosis; CHAT: choline acetyltransferase; DAPI: 4ʹ,6-diamidino-2-phenylindole; HB9: motor neuron and pancreas homeobox 1, formerly HB9; HC: healthy control; iPSC: induced pluripotent stem cell; ISL1: ISL LIM homeobox 1; Iso: isogenic control; MNs: motor neurons; TuJ: class III β-tubulin.

***Additional Figure 2:*** *Proteomic analysis of iPSC-derived motor neuron cultures.*

Additional Figure 2 Proteomic analysis of iPSC-derived motor neuron cultures.
(A) Graph showing the number of identified proteins per sample. (B) Graph showing log2 transformed data per sample. (C) Pie chart displaying the distribution of protein classes, based on PANTHER classification, for all quantified proteins (in all samples). (D) Heatmap showing expression of marker proteins for iPSCs, progenitors, neurons, motor neurons, and non-neuronal cells in addition to a few ALS-associated proteins. ALS: Amyotrophic lateral sclerosis; HC: healthy control; iPSC: induced pluripotent stem cell; Iso: isogenic control.

***Additional Figure 3:*** *Proteomic analysis of iPSC-derived motor neurons carrying ALS mutations.*

Additional Figure 3 Proteomic analysis of iPSC-derived motor neurons carrying ALS mutations.
(A) Principal component analysis of the proteomes of DIV25 MN samples, color-coded by batch (B) and replicate (R). (B) PCA after correction for batch effect of the proteomes of DIV25 MN samples, color-coded by batch (B) and replicate (R). (C) Pearson correlation analysis of the healthy control iPSC line HC-1 across batches and replicates. ALS: Amyotrophic lateral sclerosis; HC: healthy control; DIV: days *in vitro*; iPSC: induced pluripotent stem cell; Iso: isogenic control.

***Additional Figure 4:*** *Proteomic characterization of MSC-EVs.*

Additional Figure 4 Proteomic characterization of MSC-EVs.
(A) MSC-EVs were fractionated using a sucrose density gradient (1.063–1.166 M), and sucrose density was confirmed using a refractometer. Twelve individual fractions were collected, along with cell lysate (CL) as a control. Western blot analysis detected the exosomal markers CD63 and CD9 in specific fractions, while the absence of the endoplasmic reticulum protein Calnexin confirmed minimal contamination by non-EV material. (B) Nanosight Particle Tracking Analysis (five independent measurements) of MSC-EVs showing that the nominal size of MSC-EVs is 142 nm. The red color indicates SEM. (C) Gene Ontology (GO) analysis (Biological Process) based on the MSC-EV proteome. (D) Pie chart illustrating the distribution of protein classes among all quantified MSC-EV proteins, based on PANTHER classification. Data represent proteins identified from three independent MSC-EV isolations (n = 3). (E) Top 25 most abundant proteins in MSC-EVs. MSC-EVs: Mesenchymal stem cell-derived extracellular vesicles.

***Additional Figure 5:*** *MSC-EV treatment normalizes ALS proteomic changes in iPSC-derived motor neurons.*

Additional Figure 5 MSC-EV treatment normalizes ALS proteomic changes in iPSC-derived motor neurons.
(A-C) The top ten most upregulated and downregulated proteins in ALS (*C9-ALS, FUS-ALS*, and *TDP-ALS*) are shown together with the expression levels of these proteins after MSC-EV treatment. (D) Volcano plots of differentially expressed proteins in ALS iPSC-MNs (C9-ALS, FUS-ALS, TDP-ALS) treated with MSC-EVs versus untreated. Proteins significantly dysregulated in ALS versus HC are highlighted in red (upregulated) or blue (downregulated). (E) Top ten up- and downregulated proteins in ALS, with expression changes after MSC-EV treatment. ALS: Amyotrophic lateral sclerosis; iPSC: induced pluripotent stem cell; MSC-EVs: mesenchymal stem cell-derived extracellular vesicles.

***Additional Figure 6:*** *MSC-EV treatment normalizes Casp-3 levels in C9-ALS iPSC-derived motor neurons.*

Additional Figure 6 MSC-EV treatment normalizes Casp-3 levels in C9-ALS iPSC-derived motor neurons.
Immunofluorescence microscopy of Casp-3 recombinant protein (red) with Dapi counterstained nuclei (blue) in HC-1 MNs, C9-ALS-MNs (C9-1), and C9-ALS-MNs treated with MSC-EVs. Scale bar: 100 μm. Quantitative analysis of Cas-3 positive MNs normalized against the total number of cells in the image. ^****^P < 0.0001. ALS: Amyotrophic lateral sclerosis; Casp-3: Caspase-3; Dapi: 4ʹ,6-diamidino-2-phenylindole; HC: healthy control; iPSC: induced pluripotent stem cell; MNs: motor neurons; MSC-EVs: mesenchymal stem cell-derived extracellular vesicles; ns: not significant.

***Additional Table 1:*** *Information of patient and control lines used for this study.*

***Additional Table 2:*** *qPCR primers used in this study.*

***Additional Table 3:*** *ALS-mutation-specific alterations.*

***Additional Table 4:*** *ALS general alterations.*

## Data Availability

*All mass spectrometry proteomics data have been deposited to the ProteomeXchange Consortium via the PRIDE partner repository with the data set identifier PXD034824. Username: reviewer_pxd034824@ebi.ac.uk. Password: IDoEOWRj.*
